# Genome-Enabled Estimates of Additive and Nonadditive Genetic Variances and Prediction of Apple Phenotypes Across Environments

**DOI:** 10.1534/g3.115.021105

**Published:** 2015-10-22

**Authors:** Satish Kumar, Claire Molloy, Patricio Muñoz, Hans Daetwyler, David Chagné, Richard Volz

**Affiliations:** * The New Zealand Institute for Plant & Food Research Limited, Hawke’s Bay Research Center, Havelock North, 4130 New Zealand; † Agronomy Department, University of Florida, Gainesville, Florida 32611; ‡ AgriBio Center, La Trobe University, Bundoora, Victoria 3083, Australia; § Plant & Food Research, Palmerston North Research Center, Palmerston North, 4442, New Zealand

**Keywords:** GBLUP, genomic prediction, apple breeding, heritability, *Malus × domestica*

## Abstract

The nonadditive genetic effects may have an important contribution to total genetic variation of phenotypes, so estimates of both the additive and nonadditive effects are desirable for breeding and selection purposes. Our main objectives were to: estimate additive, dominance and epistatic variances of apple (*Malus × domestica* Borkh.) phenotypes using relationship matrices constructed from genome-wide dense single nucleotide polymorphism (SNP) markers; and compare the accuracy of genomic predictions using genomic best linear unbiased prediction models with or without including nonadditive genetic effects. A set of 247 clonally replicated individuals was assessed for six fruit quality traits at two sites, and also genotyped using an Illumina 8K SNP array. Across several fruit quality traits, the additive, dominance, and epistatic effects contributed about 30%, 16%, and 19%, respectively, to the total phenotypic variance. Models ignoring nonadditive components yielded upwardly biased estimates of additive variance (heritability) for all traits in this study. The accuracy of genomic predicted genetic values (GEGV) varied from about 0.15 to 0.35 for various traits, and these were almost identical for models with or without including nonadditive effects. However, models including nonadditive genetic effects further reduced the bias of GEGV. Between-site genotypic correlations were high (>0.85) for all traits, and genotype-site interaction accounted for <10% of the phenotypic variability. The accuracy of prediction, when the validation set was present only at one site, was generally similar for both sites, and varied from about 0.50 to 0.85. The prediction accuracies were strongly influenced by trait heritability, and genetic relatedness between the training and validation families.

The main goal of most fruit breeding programs is to identify the best performing individuals (or genotypes) as potential commercial cultivars, and the secondary goal is to identify genotypes that can be used as parents in future crosses. The genetic value (GV), which includes both additive and nonadditive genetic effects, predicts a genotype’s performance for a trait when asexually propagated plants are used for production (White *et al.* 2007). Nonadditive genetic variation results from interactions between alleles, whereby interactions between alleles at the same locus are called dominance, and interactions between alleles at different loci are called epistasis. When nonadditive genetic effects are an important source of variation, the use of additive genetic value or breeding value (BV) to determine potential commercial cultivars may lead to the selection of genotypes that do not have the highest GV.

In best linear unbiased prediction (BLUP) of BVs, information from performance of relatives (*e.g.*, half-sibs, full-sibs) is incorporated through the use of the additive relationship matrix derived from pedigree records (Henderson 1975). This matrix represents the expected parental contributions (*i.e.*, genome sharing) between individuals in the pedigree. Nonadditive performance of relatives could also be included in BLUP analysis through nonadditive relationship matrices derived from the pedigree. However, partitioning of total genetic components into additive and nonadditive parts, using standard pedigree-based models, requires specific family structures involving mating designs (*e.g.*, factorial, diallel). Furthermore, clonally replicated progeny trials enable the estimation of individual-level BV and GV, as well as the magnitude of genotype-by-environment interaction (G × E) (Burdon and Shelbourne 1974). BLUP analysis of such trials can provide ranking for the selection of potential parents using additive effects (BV), and potential commercial cultivars from combining predicted additive and nonadditive effects (*i.e.*, GV).

DNA marker-based relationship coefficients are more accurate than pedigree-based relationships because they account for deviations from expected parental contributions due to Mendelian sampling or segregation distortion. Furthermore, genomic relationship matrices (GRM) are particularly advantageous when pedigree relationships are not available for some individuals and/or when the recorded pedigree information may contain errors (Muñoz *et al.* 2014a; Speed and Balding 2015). Studies on animal and plant species have demonstrated that the accuracy of artificial selection can be increased by using GRM rather than pedigree-based relationship matrices (Hayes *et al.* 2009; Lee *et al.* 2010; Kumar *et al.* 2013). Numerous approaches have been developed to derive GRM based on genome-wide single nucleotide polymorphic (SNP) data (Van Raden 2008; Legarra *et al.* 2009). Similarly to the additive GRM, approaches to derive SNP-based estimates of nonadditive relationships (*e.g.*, dominance, epistasic) have also been developed (Su *et al.* 2012; Sun *et al.* 2014).

An approach termed genomic BLUP (GBLUP), which directly estimates genomic breeding values (GEBV) using additive GRM, is now commonly used in genomic selection (GS) studies of animal and plant species (Hayes *et al.* 2009; Daetwyler *et al.* 2010; Heslot *et al.* 2012; Lin *et al.* 2014). GBLUP has been shown to be equivalent to ridge regression BLUP (Goddard 2009; Stranden and Garrick 2009), and one of the advantages of GBLUP is that the model can use the well-known mixed models framework similar to the traditional pedigree-based BLUP models. Also, GBLUP can be extended to include additive and nonadditive genetic effects to potentially increase the accuracy of estimates when nonadditive genetic effects make a substantial contribution to the genetic variation (Su *et al.* 2012; Muñoz *et al.* 2014b; Sun *et al.* 2014).

Although numerous studies on estimation of genetic parameters of apple fruit phenotypes have been conducted (Durel *et al.* 1998; Alspach and Oraguzie 2002; Kouassi *et al.* 2009; Kumar *et al.* 2011), there has not yet been a systematic dissection of genetic variance of apple phenotypes into additive and nonadditive variances. The objectives of this study were threefold. The first was to estimate additive, dominance, and epistatic variances using SNP-based relationship matrices. The second was to compare the accuracy of genomic predictions using GBLUP models with or without including nonadditive genetic effects. The third was to evaluate genotype-by-environment interaction (G × E) and its impact on prediction accuracy.

## Materials and Methods

### Genetic material

A field trial consisting of 17 full-sib apple (*Malus × domestica* Borkh.) families, involving 25 advanced selections as parents, was used as the source of budwood for this study. Based on pedigree records, the average coefficient of relationship between the 25 parents was 0.15 (Supporting Information, File S1), resulting in varying degrees of relationships between the 17 full-sib families. Budwood from each of the 255 randomly selected genotypes (15 from each of the 17 families) was collected in the winter (June and July) of 2010. This budwood was held in cold storage before chip-budding eight lateral buds from each of the 255 genotypes onto nursery-grown 1-year-old ‘M9’ rootstocks in spring (September 2010), making eight clonal replicates of each of the 255 genotypes. In the following winter (July 2011), all successfully propagated seedlings were planted (3.0 × 0.5 m spacing) at two orchard sites, one in each of the key apple production regions in New Zealand, namely Hawkes Bay (HB, lat. 39°40´S, long. 176°53´E) and Motueka (MOT, lat. 41°6´S, long. 172°58´E). Generally, HB is warmer during winter, spring, and autumn than MOT. Rainfall and humidity are higher in MOT than in HB, particularly during spring. Comparing the soil types of the two sites, MOT has a more fertile soil than the HB orchard, thus trees are more vigorous at the former site. The initial goal was to plant four replicates of each genotype at each of the two sites; however, because of problems in the propagation survival, the number of plants available for each genotype varied. Ninety percent of the genotypes were represented by at least three clonal replicates at each site. There were only five individuals with only one replicate at each site. All trees received standard commercial management for nutrition, pesticide, fruit hand-thinning, and irrigation.

### Phenotypes

Fruit harvesting, which was carried out in the fruiting season (February–May) during 2013 and 2014, began when fruit from each tree were judged to be mature, based on a change in skin background color from green to yellow, and when the starch pattern index was between 2 and 3 using a scale from 0 (full starch) to 7 (no starch). Phenotypes of each individual were collected in both years. One sample of six fruit from each clonal replicate of each genotype were stored for 70 days at 0.5°, followed by a further 7 days at 20° before evaluation. Fruit firmness (FF) was determined on opposite sides of each fruit after peel removal using a Fruit Texture Analyzer (GÜSS) fitted with an 11-mm diameter probe tip. A list of visual, sensory, and instrumental traits assessed following Kumar *et al.* (2011) is shown in Table 1.

**Table 1:** Apple (*Malus × domestica* Borkh.) fruit mean trait values at Hawke’s Bay and Motueka sites, New Zealand

Trait (Abbreviation)	Description (Unit)	Hawke’s Bay		Motueka	
		Mean	Coefficient of Variation (%)	Mean	Coefficient of Variation (%)
Weight	Average individual fruit weight (g)	183*^a^*	23	192*^a^*	23
Greasiness*^b^*	Greasy sensation when finger touches skin	2.3*^a^*	66	3.3*^a^*	62
Firmness	Force required to puncture skinless tissue (kg/cm^2^)	8.7*^a^*	21	8.1*^a^*	25
Crispness*^b^*	Amount and pitch of sound generated when a 1 cm^3^ segment of flesh is first bitten with the front teeth	4.8*^a^*	21	4.5*^a^*	25
Juiciness*^b^*	Amount of free fluid released on chewing	4.3	19	4.1	22
Flavor intensity*^b^*	Sensory perception of ‘overall’ flavor intensity	3.7	9	3.6	11

a Site means were significantly (*P* < 0.01) different.

b Scoring from 0 (lowest) to 9 (highest).

### SNP genotyping

A total of 247 (out of 255) individuals were genotyped using the IRSC apple 8K SNP array v1 (Chagné *et al.* 2012), based on the Infinium II technique. Genomic DNA (gDNA) was extracted from leaves using the NucleoSpin Plant II kit (Macherey-Nagel, Düren, Germany), and quantified using the Quant-iT PicoGreen Assay (Invitrogen); 200 ng of gDNA were used as a template for the reaction, following the manufacturer’s instructions. SNP genotypes were scored using the Genotyping Module (version 1.8.4) of the Illumina GenomeStudio software (Illumina Inc.). The reliability of each genotype call was measured using the GenCall score set at a minimum of 0.15, which is a lower bound for calling genotypes relative to its associated cluster. SNPs were subsequently discarded using the following sequence of criteria: GenCall score at the 50% rank (50% GC) < 0.40; cluster separation (ClusterSep) < 0.25; more than 5% missing calls. SNPs with minor allele frequency < 0.05 were discarded, and then segregation discrepancy was checked within families. A total of 2828 SNPs were retained after various quality checks. BEAGLE 3.1 software (Browning and Browning 2007), with default settings, was then used for imputing missing SNP genotypes.

### Genomic BLUP model

A two-step approach was used to estimate genetic parameters and make genomic predictions. The first step consisted of calculating best linear unbiased estimates (BLUEs) to account for fixed effects such as replicates and year, so that each individual had a single phenotypic value for each trait at each site. These estimates were then used as ‘phenotypes’ for estimation of variance components and BLUP of additive, dominance, and epistatic effects using the following linear mixed model:

$$y=\mathrm{Xb}+Z_{1}a+Z_{2}d+Z_{3}i+Z_{4}t_{1}+Z_{5}t_{2}+Z_{6}t_{3}+e$$ (1)

where ***y*** is a vector (*ns* × 1) of adjusted phenotypes (BLUEs) on a trait; *n* is the number of individuals (247) and *s* is the number of sites (2); ***b*** is a vector of fixed effect (*i.e.*, the intercept, site); ***a*** ∼ *N*(0, ***G****_a_${\text{σ}}_{a}^{2}$*), ***d*** ∼ *N*(0, ***G****_d_*${\text{σ}}_{d}^{2}$), and ***i*** ∼ *N*(0, ***G****_aa_${\text{σ}}_{aa}^{2}$*) are the vectors (*n* × 1) of random additive, dominance, and additive-by-additive epistatic effects respectively; ***G****_a_*, ***G****_d_*, and ***G****_aa_* are the additive, dominance, and epistatic relationship matrices (*n* × *n*) respectively; $\sigma_{a}^{2}$, $\sigma_{d}^{2}$, and $\sigma_{aa}^{2}$ represent additive, dominance, and epistatic variance respectively; ***e*** ∼ *N*(0, ***I${\text{σ}}_{e}^{2}$***) is a vector of random residual terms and ***I*** is an identity matrix (*ns* × *ns*); ***t***_1_ ∼ *N*(0, ***I***_s_ ⊗ $G_{a}{\text{σ}}_{as}^{2}$), ***t***_2_ ∼ *N*(0, ***I***_s_ ⊗ $G_{d}{\text{σ}}_{ds}^{2}$), and ***t***_3_ ∼ *N*(0, ***I****_s_* ⊗ $G_{aa}{\text{σ}}_{aas}^{2}$) are the vectors (*ns* × 1) of random interactions of ***a***, ***d***, and ***i*** with site, respectively; ***I****_s_* represents an identity matrix with order equal to the number of sites; ⊗ denotes the Kronecker product operation; ***Z***_1_ – ***Z***_3_ are incidence matrices (*ns* × *n*) for the main genetic effects, and ***Z***_4_ – ***Z***_6_ are incidence matrices (*ns* × *ns*) of interaction of main genetic effects with site, respectively.

The genomic relationship matrices (***G****_a_*, ***G****_d_*, and ***G****_aa_*) were constructed using SNP marker information according to methods from previous studies (Vanraden 2008; Su *et al.* 2012). Briefly, ***G****_a_* = $\frac{\mathrm{MM}\prime }{\sum^{\text{​}}2p_{i}q_{i}}$, where ***M*** is an n × m matrix (n = number of individual, m = number of SNP loci) representing genotypes at each locus. The coefficient of *i*^th^ column of the ***M*** matrix are (0–2*p_i_*), (1–2*p_i_*) and (2–2*p_i_*) for genotypes AA, AB and BB respectively; *p_i_* and *q_i_* are the frequencies of allele A and B at *i*^th^ SNP locus, respectively. Similarly, ***G****_d_* = $\frac{HH\prime }{\sum^{\text{​}}2p_{i}q_{i}(1-2p_{i}q_{i})}$, where *H* is a n × m matrix of heterozygosity coefficients with elements (0–2*p_i_q_i_*) and (1–2*p_i_q_i_*) for homozygous (AA, BB) and heterozygous (AB) individuals at *i*^th^ SNP locus, respectively. The epistatic genomic relationship matrix (***G****_aa_*) was obtained as G#G, where # denotes the Hadamard product operation (Su *et al.* 2012). Pedigree-based relationship matrices, accounting for genetic relationships between the 25 parents, were also used for comparison purposes. Equation 1 was implemented in software ASReml v3.0 (Gilmour *et al.* 2009).

In the subsequent text, Equation 1 will be termed as Model ADE, which includes three genetic components (*a*, *d*, and *i*). BLUP-GV (= *a* + *d* + *i*) of all 247 individuals were obtained from Equation 1 using all available data. Estimates of variance components derived from Equation 1 were used for calculating narrow-sense (*h*^2^) and broad-sense (*H*^2^) heritability, as the ratio of additive (${\text{σ}}_{a}^{2}$) to phenotypic variance (sum of all variance components in the model), and the ratio of total genetic variance (${\text{σ}}_{G}^{2}$ = ${\text{σ}}_{a}^{2}$ + ${\text{σ}}_{d}^{2}$ + ${\text{σ}}_{aa}^{2}$) to phenotypic variance, respectively. The method of Yamada (1962) was followed to calculate between-site genotypic correlations using estimates of variance components from Equation 1. Reduced forms of Equation 1, by including only the additive component (Model A), and additive and dominance components (Model AD), were also tested. Goodness-of-fit for each model (*i.e.*, with and without nonadditive genetic effects) was evaluated by the log-likelihood value. The superiority of an alternative model over an additive model, obtained using all available data, was tested using a likelihood ratio test.

### Model validation

To account for the family structure of our data, we applied a cross-validation scheme by using each full-sib family in-turn as a validation population (VP) and the remaining 16 families as a training population (TP), resulting in a 17-fold cross-validation. Prediction accuracy of a model was estimated as the correlation between genomic GV (GEGV) and the observed BLUP-GV of individuals in the VP. GEGV was defined as (*a* + *d* + *i*), (*a* + *d*), and *a* for the models ADE, AD, and A, respectively. The observed BLUP-GVs were linearly regressed on the GEGV, where the regression coefficient reflected the degree of bias of the GEGV, and a regression coefficient of one indicated no bias.

Two different validation strategies were used to address practical situations. The first strategy mimics a scenario where a set of individuals (*i.e.*, VP) have not been field tested at any of the two sites (HB and MOT) of this study, *i.e.*, individuals in the VP were treated as missing at both sites. Thus, the prediction accuracy of a model in the first validation strategy was based solely on the performance of individuals in the TP. The second validation strategy considers a scenario where individuals in the VP are tested at one site (say, HB) but missing at the other site (say, MOT). The prediction accuracy in this strategy relies on the performance of TP in the target environment (MOT) and the performance of all individuals (VP and TP) from the test site (HB). This process was repeated so that all 17 families at both sites were predicted.

### Data availability

File S1 contains pedigree relationships between parents, and File S2 and File S3 contain phenotypic and genotypic data respectively.

## Results

### Basic features of the population

The average fruit weights (WT) at the HB and MOT sites were 183 g and 192 g, respectively, with a coefficient of variation (CV) of 23% at both sites (Table 1). Statistically significant differences between the site averages were observed for fruit weight (WT), greasiness (GRE), fruit firmness (FF), and crispness (CRI). The CVs were high and similar at both sites suggesting that heterogeneity of trait expression was consistent across the two key apple production sites. The average GRE score was higher but FF and CRI were significantly lower at MOT than at HB (Table 1).

**Figure 1:**
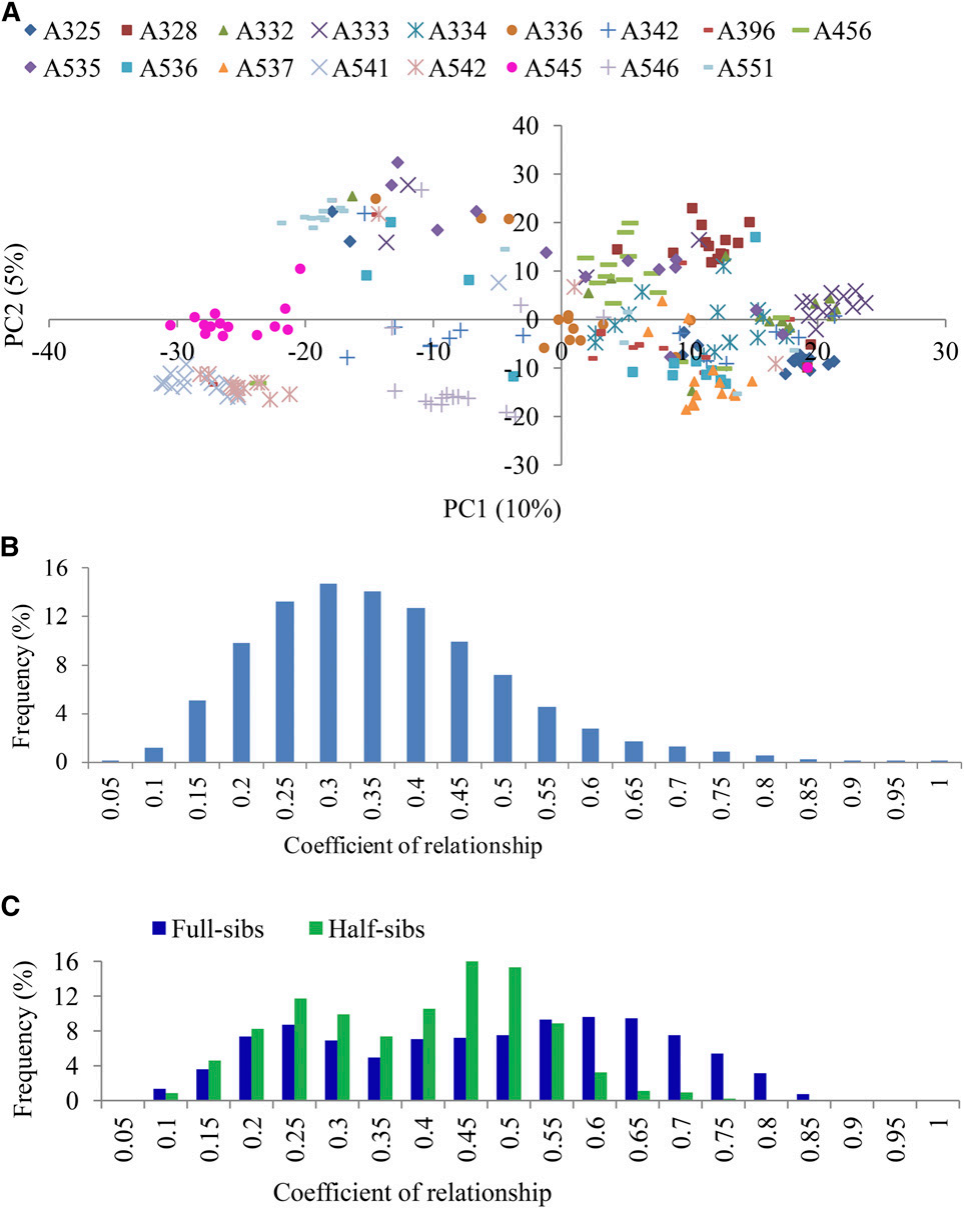
Principal coordinates (PC) plot of all 247 individuals in the apple (*Malus × domestica* Borkh.) families tested, derived from their marker genotypes (A). Pedigree-based grouping (*i.e.*, full-sib families) is also depicted in different marker colors/shape (A). Distribution of the single nucleotide polymorphism (SNP)-based coefficients of relationships between all pairs of 247 individuals (B), and for pairs of half-sibs and full-sibs is also shown (C).

A plot of the first two principal components of the SNP data matrix grouped seedlings largely according to their familial relationships (Figure 1A). In almost every family there were some individuals that did not cluster within their pedigree-assigned full-sib family groupings. Such cases were more prevalent in families such as A536, A332, A334, and A535. A distribution of the SNP-based additive coefficient of relationship between all pairs of 247 individuals is shown in Figure 1B. The SNP-based and pedigree-based average coefficients of relationship were 0.34 and 0.19, respectively. The SNP-based relatedness coefficients for pairs of supposedly half-sibs and full-sibs varied considerably (Figure 1C). The average within-family relatedness coefficient varied from 0.35 (A535) to 0.66 (A537), and the between-family relatedness coefficient varied between 0.22 and 0.50 (File S4). Results in Figure 1, A and C suggested some pollen contamination and/or mislabeling, which can be accounted for in analyses such as those in the present study by using SNP-based relationships.

### Estimates of variance components and heritability

The additive variance (${\text{σ}}_{a}^{2}$) was the major source of variability for WT (52%) and FF (50%), but the nonadditive sources were very prominent for other traits (Table 2 and File S5). On average (across traits), the additive, dominance, and epistatic effects contributed about 30%, 16%, and 19% to the total phenotypic variance. The pedigree-based average estimates of additive and nonadditive variance were 22% and 25%, respectively (results not shown). For various traits, the magnitude of variance due to interaction of genetic effects with site varied between 0 and 3%, 0 and 4%, and 0 and 8% for additive, dominance, and epistatic effects, respectively. Estimates of between-site genotypic correlation varied between 0.87 (GRE) and 0.95 (WT), and the interaction of genetic effects with site collectively accounted for less than 10% of the total phenotypic variance (Table 2).

**Table 2:** Estimates of additive ($\sigma_{a}^{2}$), dominance ($\sigma_{d}^{2}$), and epistatic ($\sigma_{\mathrm{aa}}^{2}$) genetic variance and their interaction variance in apple (*Malus × domestica* Borkh.) families with site ($\sigma_{\mathrm{as}}^{2}$, $\sigma_{\mathrm{ds}}^{2}$, $\sigma_{\mathrm{aas}}^{2}$, respectively), expressed as the percentage of phenotypic variance (defined as the sum of variance components in the model), obtained using Equation 1 (Model ADE)

Source	WT	GRE	FF	CRI	JUI	FIN
${\text{σ}}_{a}^{2}$	51.79	26.52	49.63	23.39	18.05	10.67
${\text{σ}}_{d}^{2}$	6.70	22.86	19.99	23.37	23.78	0.00
${\text{σ}}_{aa}^{2}$	18.38	23.64	9.15	19.17	18.58	23.41
${\text{σ}}_{as}^{2}$	2.31	1.08	1.32	3.38	0.00	0.80
${\text{σ}}_{ds}^{2}$	2.02	1.72	4.36	0.00	0.00	0.95
${\text{σ}}_{aas}^{2}$	0.00	8.01	0.00	0.00	4.57	3.02
${\text{σ}}_{e}^{2}$	18.80	16.17	15.56	30.70	35.02	61.16
*h*^2^	0.52	0.27	0.50	0.23	0.18	0.11
*H*^2^	0.77	0.73	0.79	0.66	0.60	0.34
*r_B_*	0.95	0.87	0.93	0.95	0.93	0.88

Estimates of narrow-sense heritability (*h*^2^), broad-sense heritability (*H*^2^), and between-site genotypic correlation (*r_B_*) are also shown for various traits. WT: fruit weight; GRE: greasiness; FF: fruit firmness; CRI: crispness; JUI: juiciness; FIN: flavor intensity.

**Figure 2:**
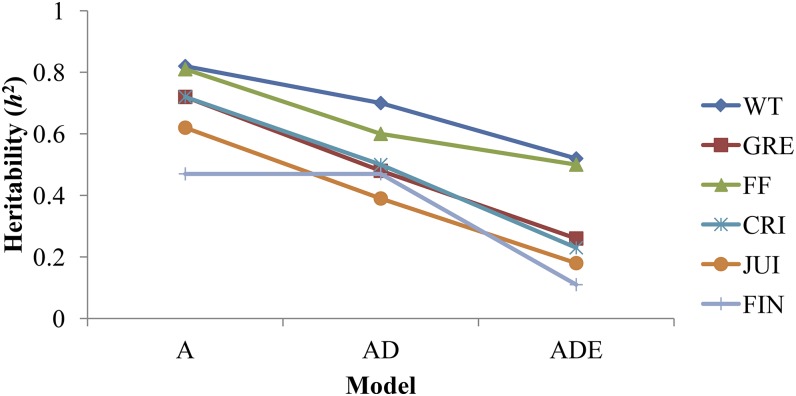
Changes in estimated heritability of various apple (*Malus × domestica* Borkh.) traits by fitting various genetic effects in the model. A, additive; AD, additive and dominance; ADE, additive, dominance, and epistatic; CRI: crispness; FF: fruit firmness; FIN: flavor intensity; GRE: greasiness; JUI: juiciness; WT: fruit weight.

The narrow-sense heritability (*h*^2^) estimate was low (< 0.20) for JUI and FIN, moderate (0.20 – 0.40) for GRE and CRI, and high (>0.40) for WT and FF (Table 2). Estimated broad-sense heritability (*H*^2^) was 0.34 for FIN and >0.60 for other traits, reflecting a substantial amount of nonadditive genetic variance. Figure 2 shows that the estimated *h*^2^ from different models varied between 0.39 and 0.70 when the epistatic effect was dropped (*i.e.*, model AD), and between 0.47 and 0.82 when dominance and epistatic effects were excluded (*i.e.*, model A) from Equation 1 (see File S6 for details). These are much higher than *h*^2^ estimates (0.11 – 0.52) of various traits obtained using the model ADE. The likelihood ratio test showed that model ADE had relatively low –2 log likelihood compared with model A (File S7), but the goodness of fit was not improved significantly (*P* > 0.05; degrees of freedom = 4).

### Accuracy of prediction

**Figure 3:**
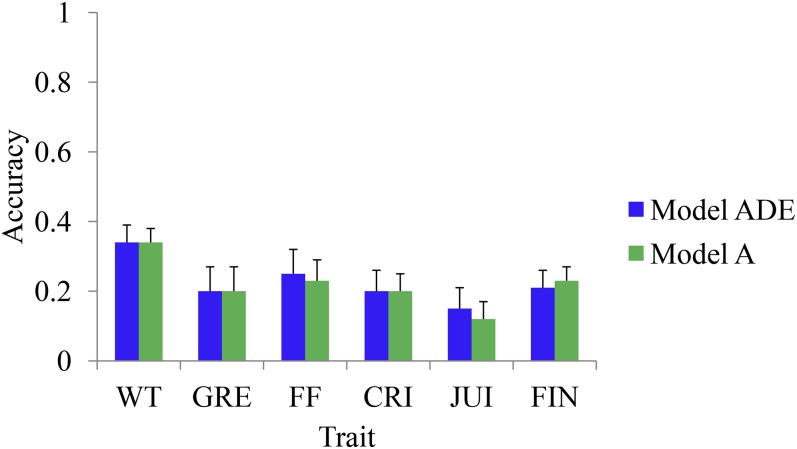
Mean (across families) prediction accuracy of various apple (*Malus × domestica* Borkh.) traits using the additive component (Model A) and both additive and nonadditive components (Model ADE). Validation samples were assumed untested at both sites. The error bars represent standard error of the mean. A, additive; ADE, additive, dominance, and epistatic; CRI: crispness; FF: fruit firmness; FIN: flavor intensity; GRE: greasiness; JUI: juiciness; WT: fruit weight.

**Figure 4:**
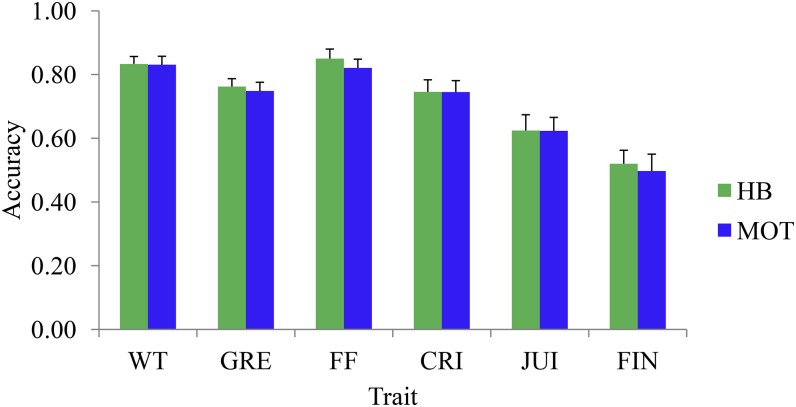
Mean (across families) prediction accuracy of various apple (*Malus × domestica* Borkh.) traits for the cross-validation strategy where validation samples are assumed untested at a particular site. The prediction model included both additive and nonadditive components (*i.e.*, model ADE). The error bars represent standard error of the mean. ADE, additive, dominance, and epistatic; CRI: crispness; FF: fruit firmness; FIN: flavor intensity; GRE: greasiness; HB, Hawke’s Bay; JUI: juiciness; MOT, Motueka; WT: fruit weight.

Prediction accuracies for the first validation strategy (no field presence of validation set) are shown in Figure 3. Correlation between the observed BLUP-GV and genomic predicted GV (*i.e.*, GEGV) was essentially the same for the additive model (model A) and for the model including additive and nonadditive effects (model ADE), and varied from about 0.15 (for JUI) to 0.35 (for FF) (Figure 3). The coefficient of regression of BLUP-GV on GEGV for various traits varied between 0.75 and 1.0 for model ADE, and between 0.55 and 0.75 for model A (File S8). These results suggested that the degree of bias is higher for the additive model than for the model ADE. For the second validation strategy (validation set present only at one site), the accuracy of predicted GEGV was generally similar for both sites, and varied from about 0.50 (for FIN) to about 0.83 (for WT and FF) (Figure 4). Prediction accuracy of validation families was moderately correlated (*r* = 0.44) with the degree of genetic relatedness to the training families (File S9). Prediction accuracy also showed strong relationship (*r* = 0.81) with trait heritability (File S10).

## Discussion

### Additive and nonadditive genetic variances

Genetic relationship matrices, constructed using genome-wide SNP markers, were fitted using a GBLUP approach to estimate nonadditive genetic variation. The difference between the pedigree-based and SNP-based additive coefficient of relationship (0.19 *vs.* 0.34) reflected that there were perhaps many more ancestral relationships not captured by the pedigree records. Different kinds of relationships (*e.g.*, half-sib, full-sib) along with permanent environmental or maternal effects shared by siblings make it challenging to partition nonadditive variance from the additive variance using traditional methods (Hill *et al.* 2008; Lee *et al.* 2010). Nevertheless, ours is the first formal attempt to estimate additive, dominance, and epistatic variances of apple phenotypes. The genome-enabled estimate of nonadditive variance was similar to the additive variance (35% *vs.* 30%), suggesting that nonadditive effects are as important as additive effects for various apple phenotypes. Mäki-Tanila and Hill (2014) showed that when heterozygosity at individual loci is high, multilocus epistasis could lead to large increases in the nonadditive part of the genotypic variance. The use of F1 families, where heterozygosity is expected to be high, could have contributed to the large nonadditive variance observed in this study. Similarly to the results of a study on loblolly pine (Muñoz *et al.* 2014b), the genome-enabled estimates of genetic variance components in this study were somewhat larger than their pedigree-based counterparts. This could be due to better capture of relatedness using genome-wide markers.

The additive variance decreased when dominance effects were included in the model, and it decreased further when epistatic effects were considered. The average *h*^2^ was 0.69, 0.52, and 0.30 for the models A, AD, and ADE, respectively (Figure 2). These results highlight the confounding nature of the additive and nonadditive effects. In other words, effects which the model ADE allocated to dominance and epistatic effects, were now being absorbed by the inferred additive genetic component in model A. This means that models ignoring nonadditive components would yield upwardly biased estimates of additive variance if the true variance components of ignored effects were not zero (Lu *et al.* 1999). The covariance between full-sibs and half-sibs includes $(\frac{1}{4}){\text{σ}}_{aa}^{2}$ and $(\frac{1}{16}){\text{σ}}_{aa}^{2}$ respectively, so there is a ‘hidden’ nonadditive component even if an additive model is fitted. Moreover, depending on the distribution of allele frequencies and linkage disequilibrium (LD) patterns, some of the variance due to interaction of alleles can manifest as additive variance (Hill *et al.* 2008; Zuk *et al.* 2012). Similarly to our results, decrease in *h*^2^ by including nonadditive effects in the model have been reported by various studies on forest trees (Waldmann *et al.* 2008; Araújo *et al.* 2012; Muñoz *et al.* 2014b).

The estimated *h*^2^ of WT (0.52) in this study was very similar to those in some other reports (Alspach and Oraguzie 2002; Kouassi *et al.* 2009). Although the *h*^2^ of FIN (0.11) was similar to that reported by Kouassi *et al.* (2009), it was lower than that found by Durel *et al.* (1998) (0.39). Heritability (*h*^2^) of CRI and JUI has been reported to vary between 0.15 and 0.40 (Durel *et al.* 1998; Alspach and Oraguzie 2002; Kouassi *et al.* 2009), but higher heritabilities (about 0.60) have been reported (Kumar *et al.* 2011). The estimated *H*^2^ in our study varied between 0.34 (FIN) and 0.79 (FF), with an average of about 0.65 (Table 2). King *et al.* (2000), using clonally replicated seedlings from a single full-sib family, reported estimates of *H*^2^ of FF, CRI, and JUI to be 0.52, 0.57, and 0.46, respectively. McKay *et al.* (2011) reported estimated *H*^2^ values of JUI and CRI as 0.72 and 0.76, respectively, which were higher than those observed in our study (0.60 and 0.66, respectively). The ratio of nonadditive (= ${\text{σ}}_{d}^{2}$+${\text{σ}}_{aa}^{2}$) to additive (${\text{σ}}_{a}^{2}$) variance varied between 0.48 (WT) and 2.35 (JUI), with an average of 1.53, suggesting that nonadditive variance contributed significantly to the expression of apple fruit phenotypes.

### Influence of explicit fitting of nonadditive components on prediction accuracy

BLUP-BV or deregressed EBVs have commonly been used as phenotypes in most applications of genomic selection in animal and plant species (Garrick *et al.* 2009; Hayes *et al.* 2009; Kumar *et al.* 2012; Resende *et al.* 2012). Such data allow only the estimation of allele substitution effects, so distinguishing between additive and nonadditive effects is not possible. The use of BLUEs as phenotypes in genomic predictions is desirable to distinguish between additive and nonadditive effects, and to reduce the computation time compared with using multiple records per individual (Su *et al.* 2012; Daetwyler *et al.* 2014; Xu *et al.* 2014). Using the phenotypes adjusted for nongenetic effects (replication, year), we obtained BLUP-GV (sum of additive, dominance, and epistatic effects) of all individuals for cross-validation of genomic predictions. There was a near-perfect correlation between BLUP-GV and BLUEs for all traits, reflecting that pedigree/family relationships had little effect on the performance ranking (*i.e.*, BLUP-GV), in contrast to unbalanced datasets with little or no clonal replication of individuals (*e.g.*, Garrick *et al.* 2009). Similar observations were reported in studies on forest tress species (Zapata-Valenzuela *et al.* 2012).

The accuracy of predicting unobserved BLUP-GV varied from about 0.15 to 0.35, and these were almost identical between the models with or without including nonadditive effects. As shown by Xu *et al.* (2014), the lack of improvement in accuracy could be attributed to the high correlation between different estimated variance components. For all traits in our study, vectors of estimated additive effects were highly correlated with nonadditive effects for both the marker-based (about 0.80) and pedigree-based (about 0.60) models. If genes are not independently distributed (*i.e.*, LD) in the parents of a population, a covariance between the additive and nonadditive effects would be introduced (Melchinger 1988), and what otherwise would be epistatic variance becomes additive or dominance variance (Hill *et al.* 2008). Studies on animal species showed that prediction accuracy increased when both (additive and nonadditive) effects rather than just additive effects were included (Su *et al.* 2012; Sun *et al.* 2014). Although the prediction accuracy of models A and ADE was similar for the apple phenotypes, the prediction bias was lower for the latter. The regression coefficients for the predictions using the model ADE were closer to 1, so including nonadditive effects did not improve the accuracy but helped to reduce the bias of predicting GV.

Relatively low prediction accuracy (Figure 3) in this study compared with that in the study by Kumar *et al.* (2012) was mainly due to the sample size (about 250 *cf*. 1000). Moreover, the large family sizes and a random cross-validation scheme in Kumar *et al.* (2012) meant that genomic predictions were essentially made within the same families that were part of the training population. For across family validation scheme as in this study, genetic relationship between the training and validation families, and population-level LD, are the key drivers of prediction accuracy (Legarra *et al.* 2008). Relationships at the level of parents and grandparents resulted in high relatedness among the 17 families in this study (File S4). Genetic relatedness between the training and validation families, and trait heritability were positively correlated with the prediction accuracy (File S9 and File S10), which is consistent with previous studies (Habier *et al.* 2007; Daetwyler *et al.* 2008).

### Genotype–environment interaction

It is common practice to include some common ‘checks’ or ‘controls’ across various test environments, to extrapolate performance of an accession at an untested site. Pedigree information could also be used for predicting performance if some known relatives were tested at the target site. Genome-wide SNPs, which provide precise estimates of genetic relationships, will provide better links between accessions at different sites for the understanding of G × E and predicting performance in different environments. The accuracy of predicting unobserved BLUP-GV at HB using performance from MOT, or vice versa, was about 0.75 for all traits except JUI (0.62) and FIN (0.52). Although the G × E variance components for JUI and FIN were similar to those for other traits (Table 2), the lower heritability of these two traits might have contributed to these results.

The prediction accuracy of the second validation strategy (Figure 4) was at least twice that of the first validation strategy (Figure 3). These results supported earlier findings that predicting the performance of untested individuals is more challenging than predicting the performance of individuals that have been evaluated at some sites (Burgueño *et al.* 2012). When the training and validation samples are observed independently over different sites and ages/years, prediction accuracies can be affected, depending on the magnitude of genotype-by-site and genotype-by-age interactions (Resende *et al.* 2012).

This study showed that nonadditive genetic effects are important sources of genetic variation for apple phenotypes, so there is a good case to account for these effects when selecting individuals as potential cultivars. For selection of individuals as parents for new crosses the additive effects is probably more relevant as the nonadditive effects do not transfer very effectively to the next generation. The GBLUP approach is relatively simple to implement to estimate additive and nonadditive variances and to predict GEBV and GEGV. GBLUP models fitting only the additive effects can predict phenotype with similar accuracy to models fitting both the additive and nonadditive effects, but the degree of bias will be lower for the latter. Also, GBLUP models ignoring nonadditive effects will provide an overestimated additive variance that will exaggerate the expected genetic gains in breeding. The high correlation between the additive and nonadditive effects may be due to the genetic relatedness between samples, so including nonadditive genetic effects in GBLUP models may still improve prediction accuracy in populations of less-related individuals—this hypothesis needs to be tested.

## Supplementary Material

Supporting Information
