# Supplementary material for: Genome-Enabled Estimates of Additive and Nonadditive Genetic Variances and Prediction of Apple Phenotypes Across Environments
Source: G3 (Bethesda). 2015 Oct 22;5(12):2711–8. doi: 10.1534/g3.115.021105 (PMC4683643; doi:10.1534/g3.115.021105)
Supplement: Supporting Information [file supp_5_12_2711__index.html]

Genome-Enabled Estimates of Additive and Nonadditive Genetic Variances and Prediction of Apple Phenotypes Across Environments — Supporting Information 

# Genome-Enabled Estimates of Additive and Nonadditive Genetic Variances and Prediction of Apple Phenotypes Across Environments

## Supporting Information for Kumar *et al.*, 2015

**Files in this Data Supplement:**

- File S4 - The average within- and between families coefficient of relationships derived from genome-wide SNPs. (.pdf, 100 KB)
- File S5 - Estimates of additive (σ2a), dominance (σ2d), epistatic (σ2aa) genetic variance and their interaction variance in apple (*Malus* x *domestica* Borkh.) families with site (σ2as, σ2ds, σ2aas, respectively) obtained using the Equation 1 (Model ADE). (.pdf, 201 KB)
- File S6 - Estimates of additive (σ2a) and dominance (σ2d) genetic variance and their interaction variance in apple (*Malus* x *domestica* Borkh.) families with site (σ2as and σ2ds respectively), expressed as the percentage of phenotypic variance (defined as the sum of variance components in the model), obtained using the Model A and Model AD. (.pdf, 201 KB)
- File S7 - -2log likelihood value for the additive model (Model A) and the model including additive and non-additive effects (Model ADE), chi-square value, and the corresponding *p*-value of likelihood ratio test (degrees of freedom = 4) for various traits ((WT: fruit weight; GRE: greasiness; FF: fruit firmness; CRI: crispness; JUI: juiciness; FIN: flavour intensity). (.pdf, 86 KB)
- File S9 - Relationship between the prediction accuracy (averaged over all traits) and genetic relationship between the training (TP) and validation (VP) families for the Model ADE. (.pdf, 82 KB)
- File S10 - Relationship between the mean (over families) accuracy and trait heritability (*h*2) for the Model ADE. (.pdf, 82 KB)
- File S8 - Coefficient of regression of observed genetic values on predicted genetic values for the additive model (Model A) and full model (Model ADE) for various fruit traits (WT: weight; GRE: greasiness; FF: firmness; CRI: crispness; JUI: juiciness; FIN: flavour intensity). (.pdf, 83 KB)
- File S1 - Parental relatedness. (.pdf, 250 KB)
- File S3 - Genotypes. (.txt, 1,382 KB)
- File S2 - Phenotypes. (.txt, 27 KB)
