# Supplementary material for: Genome-Enabled Estimates of Additive and Nonadditive Genetic Variances and Prediction of Apple Phenotypes Across Environments
Source: G3 (Bethesda). 2015 Oct 22;5(12):2711–8. doi: 10.1534/g3.115.021105 (PMC4683643; doi:10.1534/g3.115.021105)
Supplement: Supporting Information [file supp_g3.115.021105_FileS5.pdf]

**File S5 Estimates of additive ($\sigma_a^2$), dominance ($\sigma_d^2$), epistatic ($\sigma_{aa}^2$) genetic variance and their interaction variance in apple (*Malus* × *domestica* Borkh.) families with site ($\sigma_{as}^2$, $\sigma_{ds}^2$, $\sigma_{aas}^2$, respectively) obtained using the Equation 1 (Model ADE). Estimates of narrow-sense heritability ($h^2$), broad-sense heritability ($H^2$), and between-site genotypic correlation ($r_B$) are also shown for various traits (WT: fruit weight; GRE: greasiness; FF: fruit firmness; CRI: crispness; JUI: juiciness; FIN: flavour intensity). Approximate standard errors are shown in parenthesis.**

| Source | WT | GRE | FF | CRI | JUI | FIN |
|---|---|---|---|---|---|---|
| $\sigma_a^2$ | 448 (261) | 0.352 (0.395) | 1.083 (0.576) | 0.112 (0.125) | 0.019 (0.026) | 0.003 (0.005) |
| $\sigma_d^2$ | 58 (181) | 0.303 (0.309) | 0.436 (0.419) | 0.112 (0.096) | 0.025 (0.022) | 0 |
| $\sigma_{aa}^2$ | 159 (106) | 0.314 (0.168) | 0.199 (0.229) | 0.091 (0.052) | 0.019 (0.011) | 0.006 (0.003) |
| $\sigma_{as}^2$ | 20 (26) | 0.014 (0.129) | 0.029 (0.062) | 0.016 (0.017) | 0 | 0 |
| $\sigma_{ds}^2$ | 18 (28) | 0.023 (0.078) | 0.095 (0.069) | 0 | 0 | 0 |
| $\sigma_{aas}^2$ | 0 | 0.106 (0.146) | 0 | 0 | 0.005 (0.005) | 0.001 (0.004) |
| $\sigma_e^2$ | 162 (29) | 0.215 (0.265) | 0.340 (0.076) | 0.146 (0.026) | 0.037 (0.016) | 0.016 (0.007) |
| $h^2$ | 0.52 (0.24) | 0.27 (0.27) | 0.50 (0.21) | 0.23 (0.24) | 0.18 (0.23) | 0.11 (0.22) |
| $H^2$ | 0.77 (0.05) | 0.73 (0.14) | 0.79 (0.05) | 0.66 (0.07) | 0.60 (0.09) | 0.34 (0.13) |
| $r_B$ | 0.95 (0.03) | 0.87 (0.07) | 0.93 (0.04) | 0.95 (0.05) | 0.93 (0.07) | 0.88 (0.17) |
