# Supplementary material for: Genome-Enabled Estimates of Additive and Nonadditive Genetic Variances and Prediction of Apple Phenotypes Across Environments
Source: G3 (Bethesda). 2015 Oct 22;5(12):2711–8. doi: 10.1534/g3.115.021105 (PMC4683643; doi:10.1534/g3.115.021105)
Supplement: Supporting Information [file supp_g3.115.021105_FileS6.pdf]

File S6 Estimates of additive (σ_a^2) and dominance (σ_d^2) genetic variance and their interaction variance in apple (*Malus* × *domestica* Borkh.) families with site (σ_{as}^2 and σ_{ds}^2 respectively), expressed as the percentage of phenotypic variance (defined as the sum of variance components in the model), obtained using the Model A and Model AD. Estimates of narrow-sense heritability (*h*^{2}) and broad-sense heritability (*H*^{2}) are also shown for various traits (WT: fruit weight; GRE: greasiness; FF: fruit firmness; CRI: crispness; JUI: juiciness; FIN: flavour intensity).

Model A						
Source	WT	GRE	FF	CRI	JUI	FIN
σ_a^2	82.07	72.03	81.08	71.77	61.97	47.38
σ_{as}^2	2.79	5.59	2.56	2.05	0.00	1.77
σ_e^2	15.14	22.38	16.36	26.18	38.03	50.85
h^2	0.82	0.72	0.81	0.72	0.62	0.47

Model AD						
Source	WT	GRE	FF	CRI	JUI	FIN
σ_a^2	70.13	48.39	60.20	50.27	39.09	47.30
σ_d^2	12.19	25.40	21.42	23.45	25.99	0.00
σ_{as}^2	1.62	4.23	1.00	2.00	0.00	1.16
σ_{ds}^2	1.53	1.71	3.69	0.00	0.00	1.20
σ_e^2	14.54	20.26	13.69	24.28	34.92	50.34
h^2	0.70	0.48	0.60	0.50	0.39	0.47
H^2	0.82	0.74	0.82	0.74	0.65	0.47
