# Supplementary material for: Genome-Enabled Estimates of Additive and Nonadditive Genetic Variances and Prediction of Apple Phenotypes Across Environments
Source: G3 (Bethesda). 2015 Oct 22;5(12):2711–8. doi: 10.1534/g3.115.021105 (PMC4683643; doi:10.1534/g3.115.021105)
Supplement: Supporting Information [file supp_g3.115.021105_FileS7.pdf]

**File S7** -2log likelihood value for the additive model (Model A) and the model including additive and non-additive effects (Model ADE), chi-square value, and the corresponding *p*-value of likelihood ratio test (degrees of freedom = 4) for various traits ((WT: fruit weight; GRE: greasiness; FF: fruit firmness; CRI: crispness; JUI: juiciness; FIN: flavour intensity).

| Trait | Model A | Model ADE | Chi-square | *p*-value |
|---|---|---|---|---|
| WT | 3709.4 | 3705.9 | 3.5 | 0.48 |
| GRE | 679.0 | 671.0 | 8.0 | 0.09 |
| FF | 697.6 | 692.3 | 5.3 | 0.26 |
| CRI | 95.7 | 90.3 | 5.4 | 0.25 |
| JUI | -569.6 | -576.7 | 7.1 | 0.13 |
| FIN | -1172.4 | -1177.2 | 4.7 | 0.31 |
