# Supplementary material for: Genome-Enabled Estimates of Additive and Nonadditive Genetic Variances and Prediction of Apple Phenotypes Across Environments
Source: G3 (Bethesda). 2015 Oct 22;5(12):2711–8. doi: 10.1534/g3.115.021105 (PMC4683643; doi:10.1534/g3.115.021105)
Supplement: Supporting Information [file supp_g3.115.021105_FileS9.pdf]

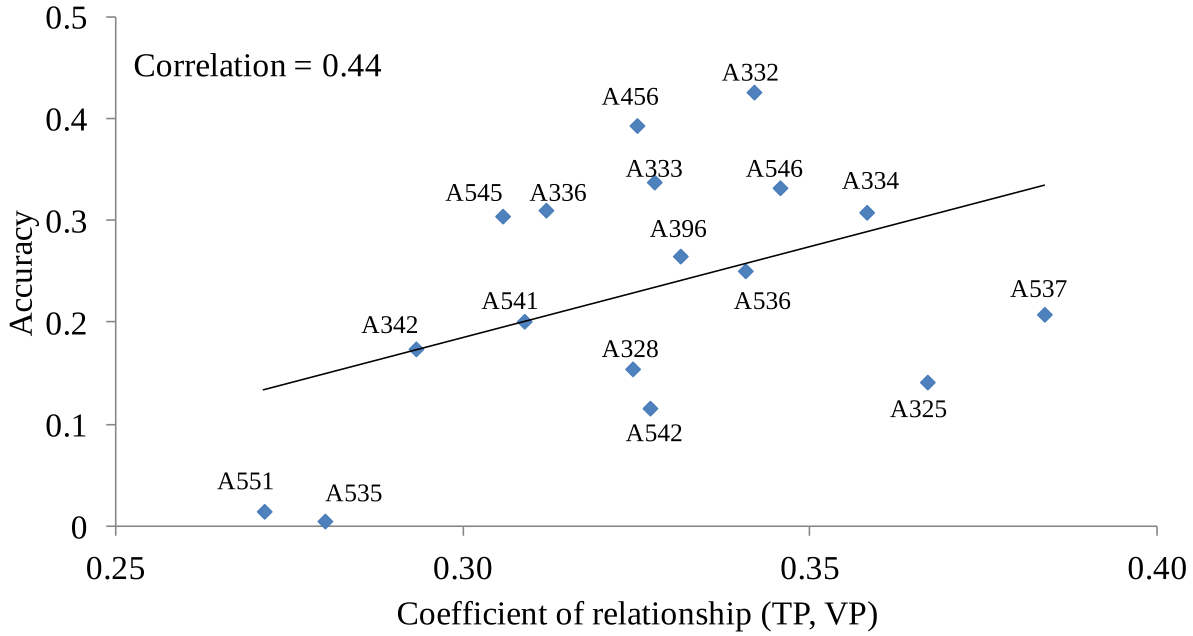

File S9. Relationship between the prediction accuracy (averaged over all traits) and genetic relationship between the training (TP) and validation (VP) families for the Model ADE. Each family was used in-turn as a validation population.
