# Supplementary material for: Genome-Enabled Estimates of Additive and Nonadditive Genetic Variances and Prediction of Apple Phenotypes Across Environments
Source: G3 (Bethesda). 2015 Oct 22;5(12):2711–8. doi: 10.1534/g3.115.021105 (PMC4683643; doi:10.1534/g3.115.021105)
Supplement: Supporting Information [file supp_g3.115.021105_FileS8.pdf]

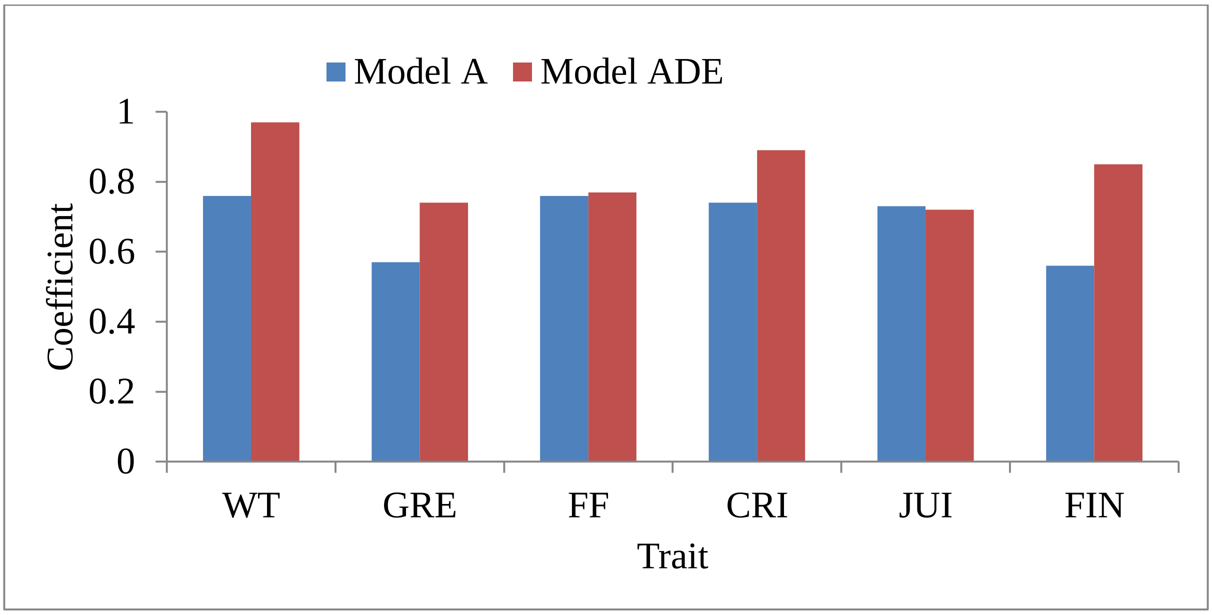

File S8. Coefficient of regression of observed genetic values on predicted genetic values for the additive model (Model A) and full model (Model ADE) for various fruit traits (WT: weight; GRE: greasiness; FF: firmness; CRI: crispness; JUI: juiciness; FIN: flavour intensity).
