# Supplementary material for: Genome-Enabled Estimates of Additive and Nonadditive Genetic Variances and Prediction of Apple Phenotypes Across Environments
Source: G3 (Bethesda). 2015 Oct 22;5(12):2711–8. doi: 10.1534/g3.115.021105 (PMC4683643; doi:10.1534/g3.115.021105)
Supplement: Supporting Information [file supp_g3.115.021105_FileS1.pdf]

Genome-enabled estimates of additive and non-additive genetic variances and prediction of apple phenotypes across environments

Satish Kumar[*,1], Claire Whitworth[*], Patricio Muñoz[§], Hans Daetwyler[†], David Chagné[‡], Richard Volz[*]

[*]The New Zealand Institute for Plant & Food Research Limited (PFR), Hawke's Bay Research Centre, Havelock North, New Zealand

[§]Agronomy Department, University of Florida, Gainesville, FL 32611, USA

[†]AgriBio Centre, La Trobe University, Bundoora 3083, Victoria, Australia

[‡]PFR, Palmerston North Research Centre, Palmerston North, New Zealand

[1]Corresponding author: Plant & Food Research Limited (PFR), Hawke's Bay Research Centre, Havelock North, New Zealand. E-mail: Satish.Kumar@plantandfood.co.nz

File S1 Genetic relationships between the 25 parents of 17 families (the cells filled with green colour).

Parent	P1	P2	P3	P4	P5	P6	P7	P8	P9	P10	P11	P12	P13	P14	P15	P16	P17	P18	P19	P20	P21	P22	P23	P24	P25
P1		0.25	0.25	0	0.25	0.25	0.25	0	0	0.25	0.5	0.5	0.125	0	0.625	0.625	0.625	0.25	0.25	0.25	0.375	0.375	0	0	0.25
P2	0.25		0.5	0.125	0.5	0.5	0.5	0	0	0.125	0.125	0.125	0.25	0	0.25	0.25	0.25	0.125	0.125	0.125	0.375	0.375	0	0	0.5
P3	0.25	0.5		0.125	0.5	0.5	0.5	0	0	0.125	0.125	0.125	0.25	0	0.25	0.25	0.25	0.125	0.125	0.125	0.625	0.625	0	0	0.5
P4	0	0.125	0.125		0.125	0.125	0.125	0	0	0	0	0	0.0625	0	0	0	0	0	0	0	0.0625	0.0625	0	0	0.125
P5	0.25	0.5	0.5	0.125		0.5	0.5	0	0	0.125	0.125	0.125	0.25	0	0.25	0.25	0.25	0.125	0.125	0.125	0.375	0.375	0	0	0.5
P6	0.25	0.5	0.5	0.125	0.5		0.5	0	0	0.125	0.125	0.125	0.25	0	0.25	0.25	0.25	0.125	0.125	0.125	0.375	0.375	0	0	0.5
P7	0.25	0.5	0.5	0.125	0.5	0.5		0	0	0.125	0.125	0.125	0.25	0	0.25	0.25	0.25	0.125	0.125	0.125	0.375	0.375	0	0	0.5
P8	0	0	0	0	0	0	0		0	0	0	0	0	0	0	0	0	0	0	0	0	0	0	0	0
P9	0	0	0	0	0	0	0	0		0	0	0	0	0	0	0	0	0	0	0	0	0	0.5	0.5	0
P10	0.25	0.125	0.125	0	0.125	0.125	0.125	0	0		0.125	0.125	0.0625	0	0.1875	0.1875	0.1875	0.125	0.125	0.125	0.1875	0.1875	0	0	0.125
P11	0.5	0.125	0.125	0	0.125	0.125	0.125	0	0	0.125		0.5	0.0625	0	0.3125	0.3125	0.3125	0.125	0.125	0.125	0.1875	0.1875	0	0	0.125
P12	0.5	0.125	0.125	0	0.125	0.125	0.125	0	0	0.125	0.5		0.0625	0	0.3125	0.3125	0.3125	0.125	0.125	0.125	0.1875	0.1875	0	0	0.125
P13	0.125	0.25	0.25	0.0625	0.25	0.25	0.25	0	0	0.0625	0.0625	0.0625		0.25	0.125	0.125	0.125	0.0625	0.0625	0.0625	0.1875	0.1875	0	0	0.25
P14	0	0	0	0	0	0	0	0	0	0	0	0	0.25		0	0	0	0	0	0	0	0	0	0	0
P15	0.625	0.25	0.25	0	0.25	0.25	0.25	0	0	0.1875	0.3125	0.3125	0.125	0		0.625	0.625	0.1875	0.1875	0.1875	0.3125	0.3125	0	0	0.25
P16	0.625	0.25	0.25	0	0.25	0.25	0.25	0	0	0.1875	0.3125	0.3125	0.125	0	0.625		0.625	0.1875	0.1875	0.1875	0.3125	0.3125	0	0	0.25
P17	0.625	0.25	0.25	0	0.25	0.25	0.25	0	0	0.1875	0.3125	0.3125	0.125	0	0.625	0.625		0.1875	0.1875	0.1875	0.3125	0.3125	0	0	0.25
P18	0.25	0.125	0.125	0	0.125	0.125	0.125	0	0	0.125	0.125	0.125	0.0625	0	0.1875	0.1875	0.1875		0.5	0.5	0.1875	0.1875	0	0	0.125
P19	0.25	0.125	0.125	0	0.125	0.125	0.125	0	0	0.125	0.125	0.125	0.0625	0	0.1875	0.1875	0.1875	0.5		0.5	0.1875	0.1875	0	0	0.125
P20	0.25	0.125	0.125	0	0.125	0.125	0.125	0	0	0.125	0.125	0.125	0.0625	0	0.1875	0.1875	0.1875	0.5	0.5		0.1875	0.1875	0	0	0.125
P21	0.375	0.375	0.625	0.0625	0.375	0.375	0.375	0	0	0.1875	0.1875	0.1875	0.1875	0	0.3125	0.3125	0.3125	0.1875	0.1875	0.1875		0.625	0	0	0.375
P22	0.375	0.375	0.625	0.0625	0.375	0.375	0.375	0	0	0.1875	0.1875	0.1875	0.1875	0	0.3125	0.3125	0.3125	0.1875	0.1875	0.1875	0.625		0	0	0.375
P23	0	0	0	0	0	0	0	0	0.5	0	0	0	0	0	0	0	0	0	0	0	0	0		0.75	0
P24	0	0	0	0	0	0	0	0	0.5	0	0	0	0	0	0	0	0	0	0	0	0	0	0.75		0
P25	0.25	0.5	0.5	0.125	0.5	0.5	0.5	0	0	0.125	0.125	0.125	0.25	0	0.25	0.25	0.25	0.125	0.125	0.125	0.375	0.375	0	0	

Family ID (corresponding to Figure 1A) and the parents involved are shown below:

Family	A325	A328	A332	A333	A334	A336	A342	A396	A456	A535	A536	A537	A541	A542	A545	A546	A551
Mother	P3	P12	P1	P1	P2	P5	P7	P6	P11	P8	P10	P10	P18	P18	P19	P19	P4
Father	P15	P16	P16	P17	P14	P15	P20	P13	P25	P2	P22	P21	P23	P24	P24	P9	P9
